# Supplementary material for: CD1d‐mediated lipid presentation by CD11c+ cells regulates intestinal homeostasis
Source: EMBO J. 2018 Jan 29;37(5):e97537. doi: 10.15252/embj.201797537 (PMC5830915; doi:10.15252/embj.201797537)
Supplement: Supplementary file 1 — Appendix [file EMBJ-37-e97537-s001.pdf]

CD1d-MEDIATED LIPID PRESENTATION BY CD11c^{+} CELLS REGULATES INTESTINAL HOMEOSTASIS

Julia Sáez de Guinoa, Rebeca Jimeno, Mauro Gaya, David Kipling, María José Garzón, Deborah Dunn-Walters, Carles Ubeda & Patricia Barral

APPENDIX FIGURES S1-S12

APPENDIX TABLES S1-S2

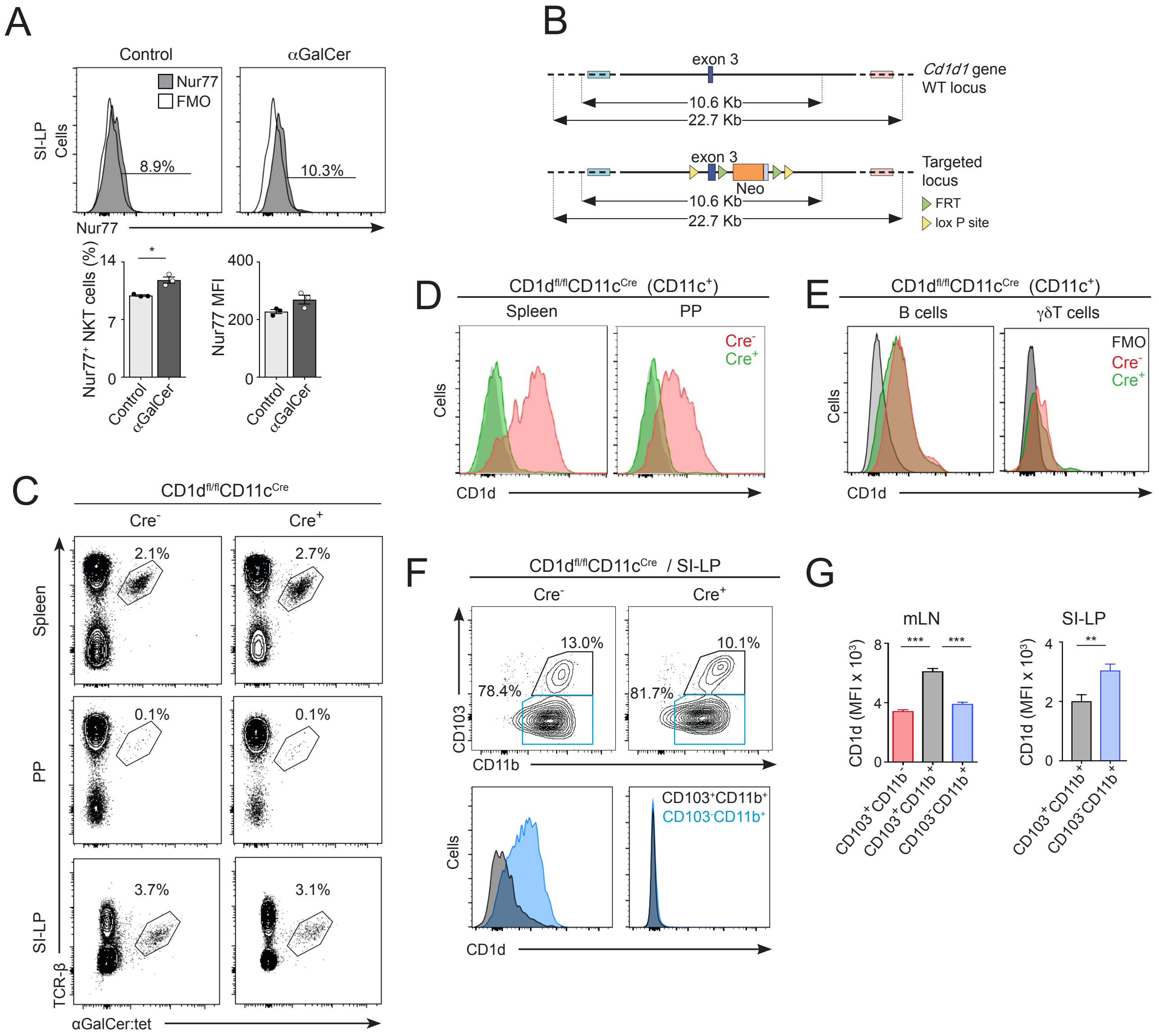

Appendix Figure S1. Characterization of conditional CD1d mice

(A) C57BL/6 mice were orally gavaged with αGalCer and Nur77 up-regulation in SI-LP iNKT cells was analysed 16h later. Flow-cytometry profiles (top), frequency Nur77⁺ iNKT cells and Nur77 MFI (bottom) are shown. Flow-cytometry profiles show Nur77 (grey histogram) or FMO (empty histogram) in iNKT cells from SI-LP of αGalCer treated mice. *$p < 0.05$, two-tailed unpaired t-test. Data are from 3 experiments

(B) Scheme showing the wild type and targeted *Cd1d1* locus containing two loxP sites flanking the exon 3 as well as two FRT sites for the removal of the Neomycin resistance cassette.

(C) Flow-cytometry analysis for iNKT cells in the depicted tissues from CD1d$^{fl/fl}$CD11c^{Cre} Cre$^-$ and Cre$^+$ mice. Numbers indicate percentage of iNKT cells from TCRβ$^+$ cells. Data represent 3-4 experiments.

(D-E) Flow-cytometry analysis for CD1d expression in DC (CD11c$^+$) cells in spleen and Peyer's patches **(D)** as well as in splenic B cells (**E**, left) and γδT cells (**E**, right) from Cre$^-$ (red) and Cre$^+$ (green) mice. Data represent 3-5 experiments.

(F-G) CD11c$^+$MHC-II$^+$ populations in the SI-LP from CD1d$^{fl/fl}$CD11c^{Cre} Cre$^-$ and Cre$^+$ mice were analysed by flow-cytometry. **(F)** Gating strategy (top) and CD1d expression (bottom) showing CD103$^+$CD11b$^+$ (black) and CD103$^-$CD11b$^+$ (blue) cells. **(G)** Main fluorescence intensity (MFI) for CD1d expression in the depicted DC/macrophage populations; ***$p<0.001$. Data are from 3 experiments

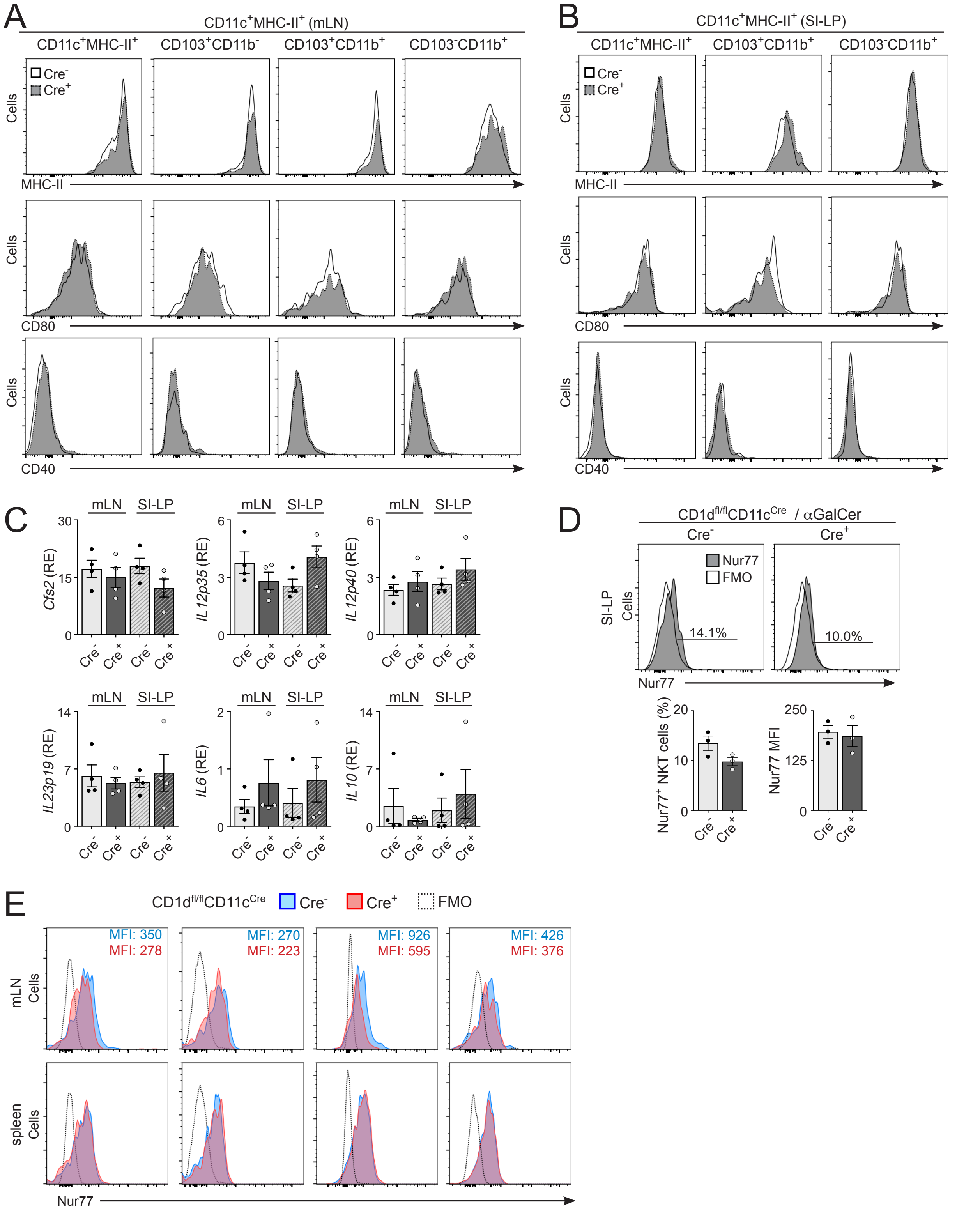

Appendix Figure S2. CD11c$^+$ cells in CD1d$^{fl/fl}$CD11c^{Cre} mice

(A-B) Expression of the depicted markers in the indicated DC/macrophage populations from the mLN **(A)** and SI-LP **(B)** of Cre$^-$ (empty histogram) or Cre$^+$ (grey) CD1d$^{fl/fl}$CD11c^{Cre} mice. Data represent 3 experiments

(C) CD11c$^+$ cells were sort purified from the mLN and SI-LP of Cre$^-$ or Cre$^+$ CD1d$^{fl/fl}$CD11c^{Cre} mice. qPCR analysis of mRNA encoding the depicted cytokines are shown (data are normalized to GAPDH. RE=relative expression)

(D) Nur77 expression on iNKT cells in the SI-LP from CD1d$^{fl/fl}$CD11c^{Cre} Cre$^-$ and Cre$^+$ mice 16 h after oral challenge with αGalCer. Flow-cytometry profiles for Nur77 expression (top), frequency of Nur77$^+$ iNKT cells and Nur77 MFI (bottom) are shown. Flow-cytometry profiles show Nur77 (grey histogram) or FMO (empty histogram) in iNKT cells from SI-LP of αGalCer treated mice. Data are from 3 experiments.

(E) Flow-cytometry profiles showing intracellular Nur77 expression in iNKT cells from mLN (top) and spleen (bottom) from CD1d$^{fl/fl}$CD11c^{Cre} Cre$^-$ (blue) and Cre$^+$ (red) littermates. Numbers indicate Nur77 MFI for Cre$^-$ (blue) and Cre$^+$ (red) mice.

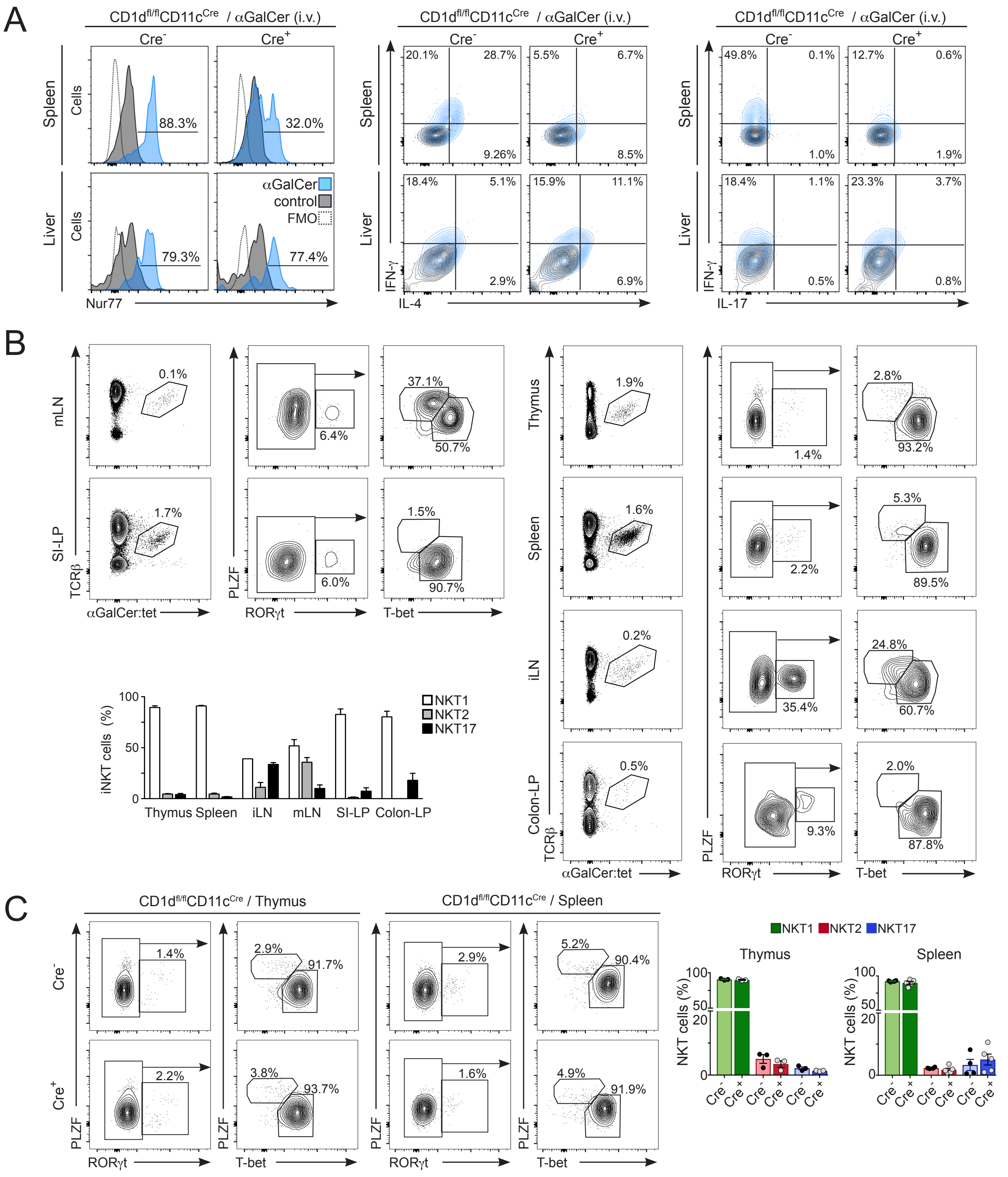

Appendix Figure S3. iNKT cell subsets in tissues.

(A) $CD1d^{fl/fl}CD11c^{Cre}$ Cre^- and Cre^+ mice were i.v. injected with αGalCer and 2 h later Nur77 expression and cytokine secretion by iNKT cells was analysed in the spleen and liver. Left panels show frequency of Nur77-expressing iNKT cells in αGalCer treated or control $CD1d^{fl/fl}CD11c^{Cre}$ Cre^- and Cre^+ mice. Right and middle panels show production of the depicted cytokines in iNKT cells from αGalCer treated (blue plots) or control (black plots) $CD1d^{fl/fl}CD11c^{Cre}$ Cre^- and Cre^+ mice. Numbers indicate percentages of iNKT cells in the depicted gates for αGalCer treated-mice.

(B) Flow-cytometry profiles showing gating strategy for iNKT cells, and NKT1 ($ROR\gamma t^- PLZF^{lo} T\text{-}bet^+$), NKT2 ($ROR\gamma t^- PLZF^{hi} T\text{-}bet^-$) and NKT17 ($PLZF^{int} ROR\gamma t^+$) cells in the depicted tissues of WT C57BL/6 mice. iNKT frequencies are referred to $TCR\beta^+$ cells. Bar graph shows frequency of NKT1, NKT2 and NKT17 cells in the indicated tissues. Data are from 3 experiments.

(C) Flow-cytometry plots (left) and quantification (right) of iNKT cell populations in the thymus and spleen from $CD1d^{fl/fl}CD11c^{Cre}$ mice. Data represent at least 3 experiments. Numbers indicate percentage of cells in the indicated gates.

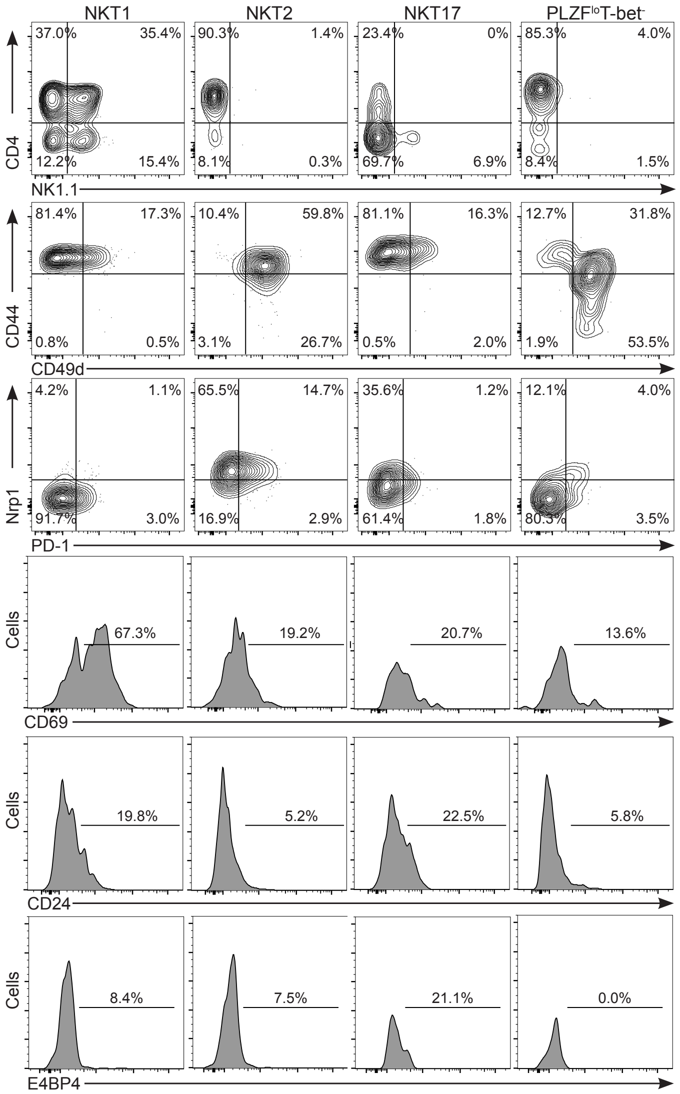

Appendix Figure S4. Characterization of mLN iNKT cells.
Flow-cytometry analysis of the indicated markers in T-bet$^-$PLZF^{lo} NKT cells from mLN. Data represent 2-4 experiments. Numbers indicate percentage of cells in the indicated gates.

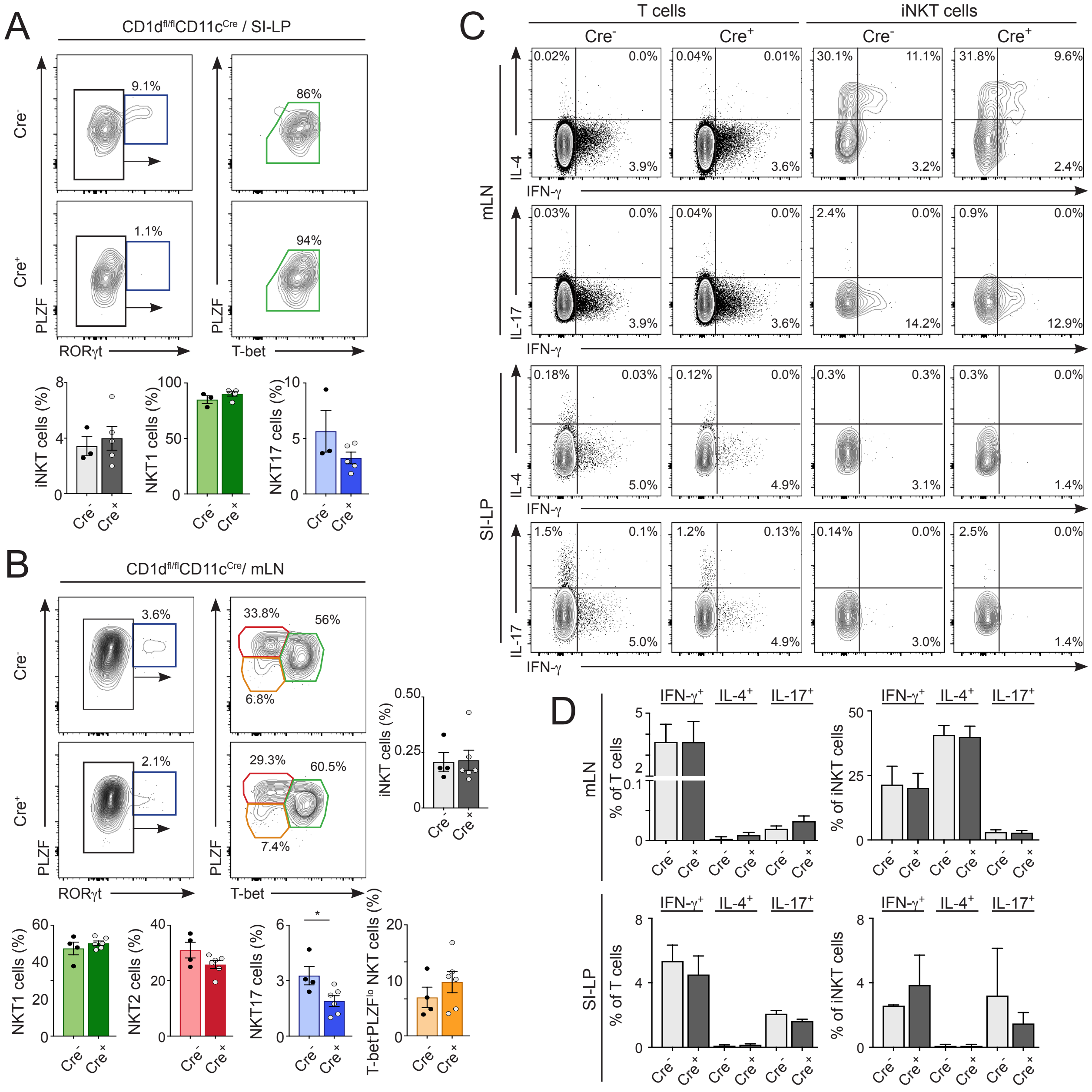

Appendix Figure S5. Characterization of intestinal iNKT cells.

(A-B) Analysis of iNKT cells populations in the SI-LP **(A)** and mLN **(B)** from 3-week old CD1d$^{fl/fl}$CD11c^{Cre} mice, showing flow-cytometry plots, frequency of total iNKT cells (grey) and NKT1 (RORγt^{-}PLZF^{lo}T-bet^{+}; green), NKT2 (RORγt^{-} PLZF^{hi}T-bet^{-}; red), NKT17 (PLZF^{int}RORγt^{+}; blue) and PLZF^{lo}T-bet^{-} NKT cells (orange). Data are from 3 experiments **(C-D)** Single cell suspension from mLN (top) or SI-LP (bottom) were prepared and stimulated for 3h with PMA/ ionomycin. Graphs show flow-cytometry plots **(C)** and frequency **(D)** of cytokine-producing T cells and iNKT cells as detected by intracellular staining. Data are from 3 experiments

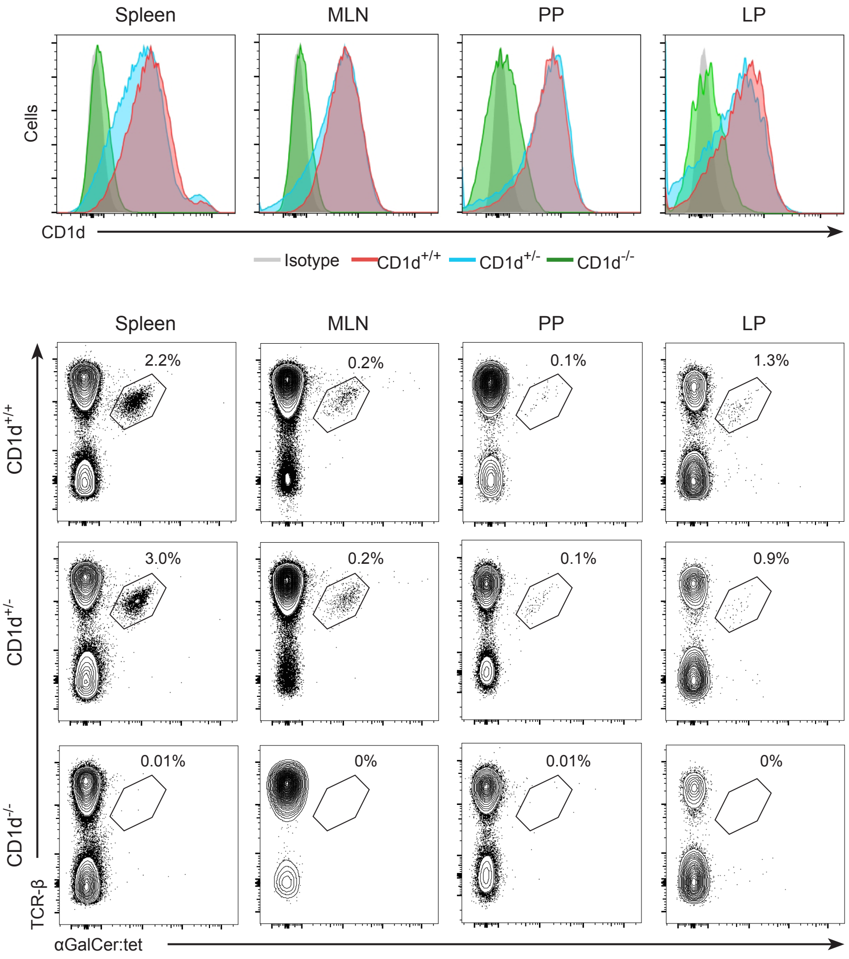

Appendix Figure S6. Characterization of CD1d-deficient mice

Flow-cytometry analysis for CD1d expression (top) and iNKT cells (bottom) in the depicted tissues from CD1d$^{+/+}$ (red), CD1d$^{+/-}$ (blue) and CD1d$^{-/-}$ (green) mice. Numbers indicate percentage of iNKT cells from TCRβ^{+} cells. Data represent 3-5 independent experiments.

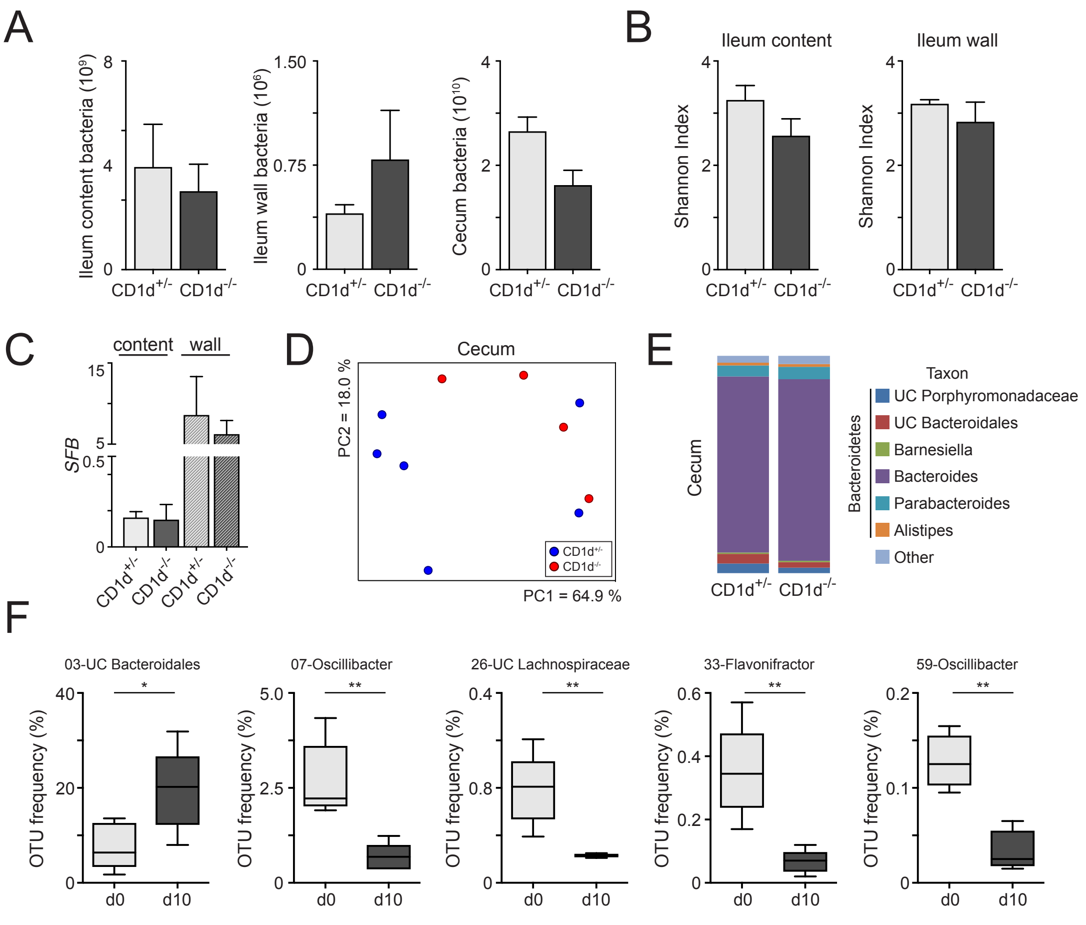

Appendix Figure S7. Bacterial load and cecal microbiota in CD1d-deficient mice.
(A) Bacterial load determined by qPCR in the ileum content, ileum wall, and cecum content from CD1d$^{+/-}$ and CD1d$^{-/-}$ mice.
(B) Shannon diversity index in the ileum content and Ileum wall from CD1d$^{+/-}$ and CD1d$^{-/-}$ mice (n=4-6)
(C) qPCR for SFB in the ileum content and wall from CD1d$^{+/-}$ and CD1d$^{-/-}$ mice (n=4-6). Data is shown as relative expression respect Eubacterial 16S rRNA.
(D) PCoA using the Yue & Clayton distances obtained among cecal samples from CD1d$^{+/-}$ and CD1d$^{-/-}$ mice. The axes show the percentage of variation explained by PC1 and PC2. Each dot corresponds to one mouse.
(E) Average relative abundance of the most frequent (>1%) genus in the cecum content from CD1d$^{+/-}$ and CD1d$^{-/-}$ mice. Taxa are shown and labelled with different colours as indicated.
(F) Relative abundance of the specified OTUs in faecal samples of C57BL/6 mice before (d0) and 10 days (d10) after αGalCer oral treatment. Line indicates the median, boxes show the 75th and the 25th percentiles, and whiskers indicate the maximum and minimum values. *p< 0.05, **p< 0.01, two-tailed Wilcoxon test.

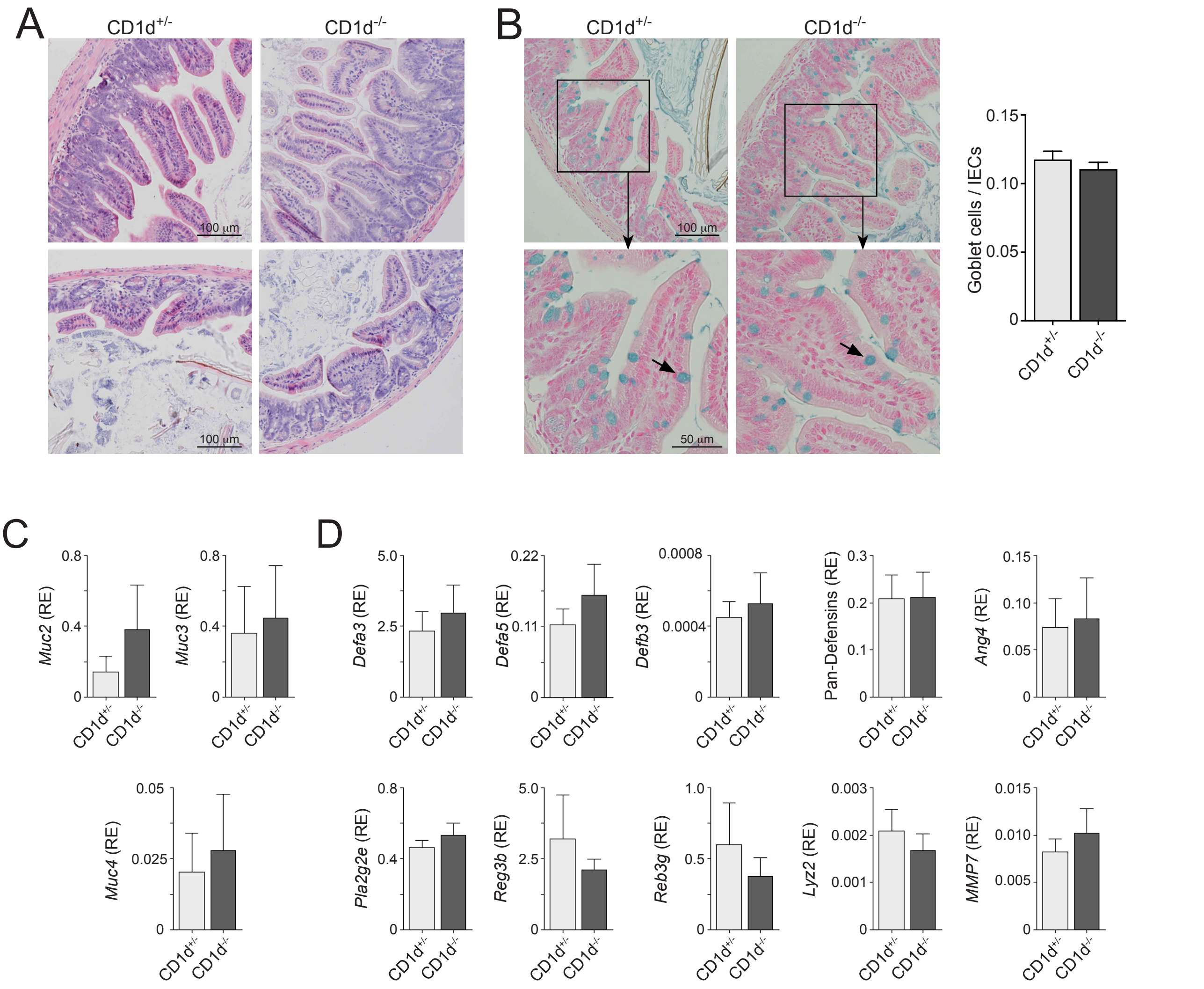

Appendix Figure S8. Intestinal histology for CD1d$^{-/-}$ mice.

(A) Hematoxylin and Eosin staining of ileum sections from CD1d$^{+/-}$ and CD1d$^{-/-}$ mice (2 experiments).

(B) Goblet cells (blue) and epithelial cells (red; left), and number of Goblet cells (referred to the number of IECs; right) on ileum sections from CD1d$^{+/-}$ and CD1d$^{-/-}$ mice. Arrows indicate Goblet cell examples.

(C-D) qPCR analysis of mRNA encoding the indicated mucin **(C)** and antimicrobial peptide **(D)** genes in FACS sorted IEC from CD1d$^{+/-}$ and CD1d$^{-/-}$ mice. Data are normalized to *GAPDH*; RE=relative expression. Data is from 3 experiments

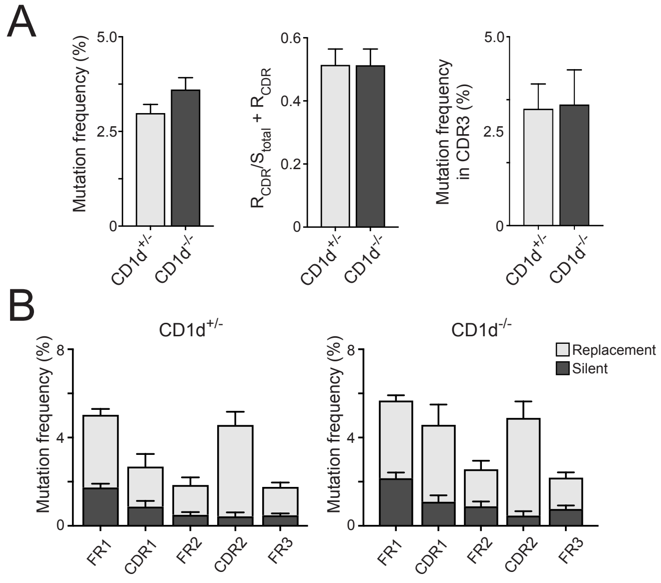

Appendix Figure S9. IgA mutation frequencies in CD1d$^{-/-}$ mice.

(A) Frequency of mutations in IgA (left), affinity maturation index (middle) and frequency of mutations in complementary-determining region 3 (CDR3; right) of IgA sequenced from CD1d$^{+/-}$ and CD1d$^{-/-}$ mice. RCDR, replacement mutations in CDR1 and CDR2; Stotal, silent mutations in CDR1-2 and in framework regions 1, 2 and 3 (FWR1-3).

(B) Frequency of replacement and silent mutations in FWRs and CDRs in IgA IGHV genes in the PP from CD1d$^{+/-}$ and CD1d$^{-/-}$ mice. (A-B) n=5-6; mean +/- SEM.

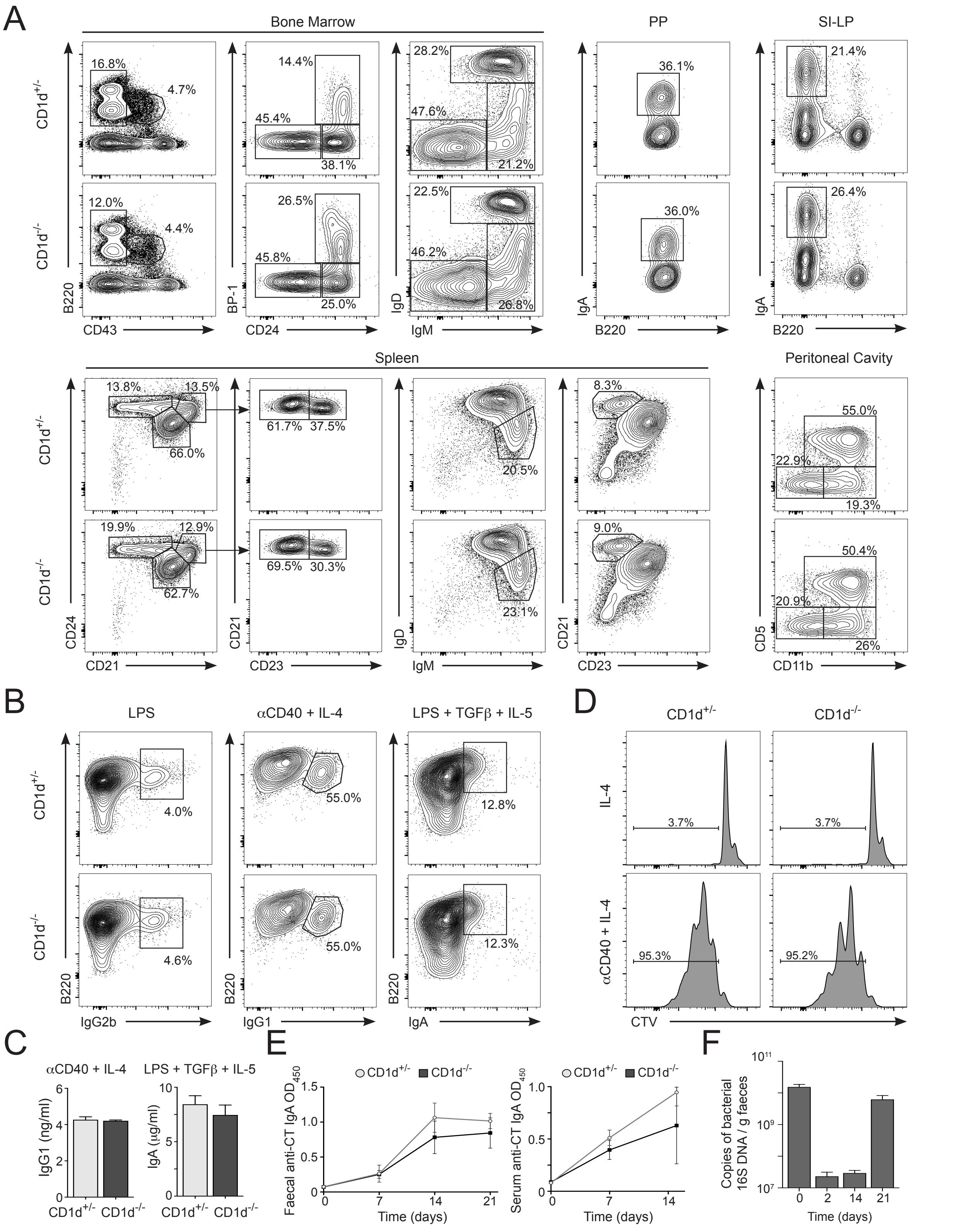

Appendix Figure S10. Characterization of B cell development and function in CD1d$^{-/-}$ mice.

(A) Flow-cytometry plots of B cell populations in bone marrow, Peyer's patches, SI-LP, spleen, and peritoneal cavity from CD1d$^{+/-}$ and CD1d$^{-/-}$ mice. Data represent 3 experiments.

(B-C) Primary B cells from CD1d$^{+/-}$ and CD1d$^{-/-}$ mice were treated with the depicted stimuli and immunoglobulin class switch was determined 5 days after by flow-cytometry **(B)** and ELISA **(C)**. Data represent 3 experiments.

(D) Proliferation of primary B cells from CD1d$^{+/-}$ and CD1d$^{-/-}$ mice 4 days after culture with the indicated stimuli. CTV stands for CellTrace Violet. Data represent 3 experiments.

(E) Cholera toxin (CT) specific IgA levels in faeces and serum of CT-immunized CD1d$^{+/-}$ and CD1d$^{-/-}$ mice

(F) Mice were orally treated with antibiotics for 14 days, and left untreated for 7 days (day 21) to allow microbiota re-covering. Bacteria load in the stool was determined by qPCR at the indicated time points (n=2-4).

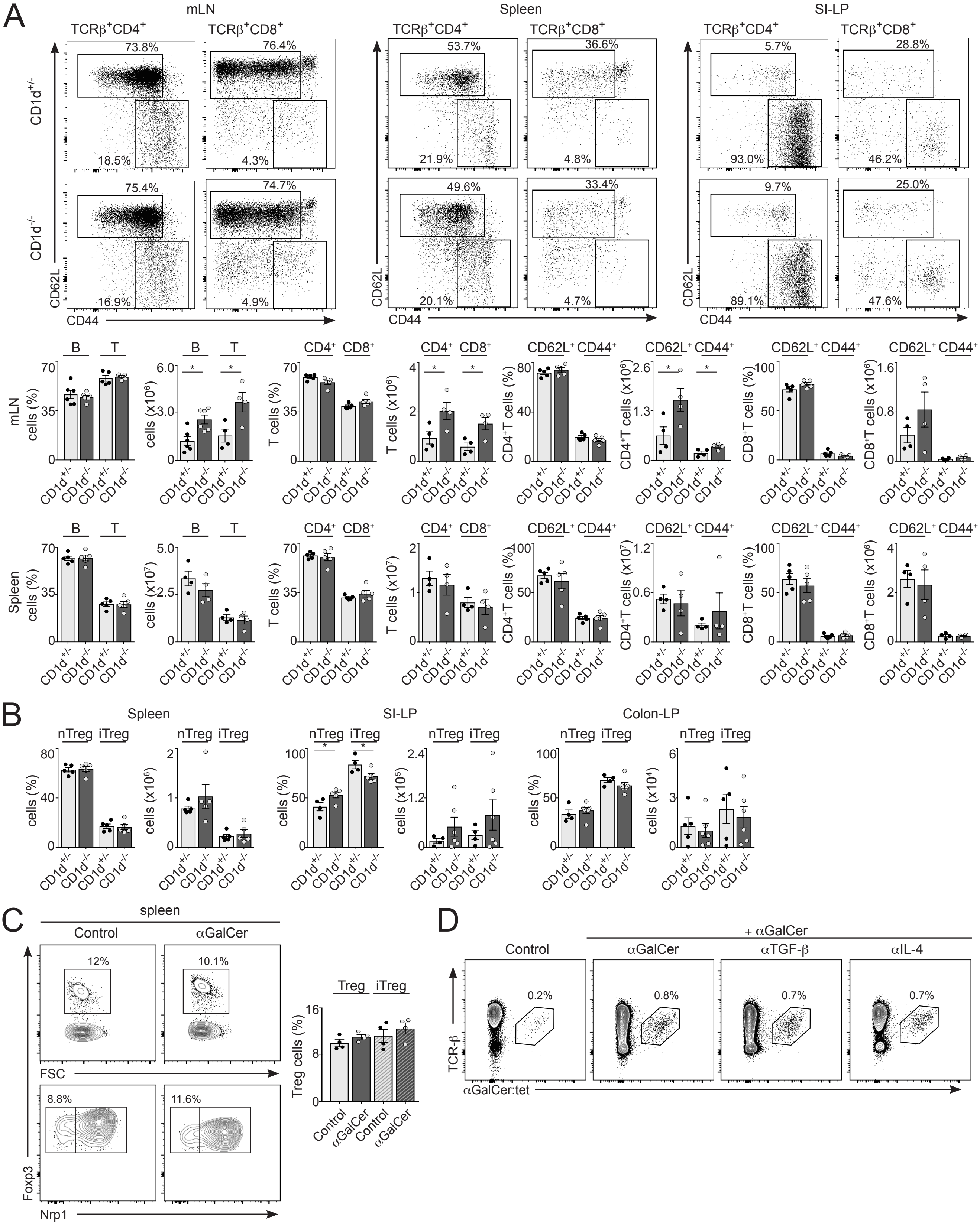

Appendix Figure S11. T cell populations in $CD1d^{-/-}$ mice

(A) Analysis of immune cell populations in the tissues from $CD1d^{+/-}$ and $CD1d^{-/-}$ mice. Flow cytometry plots (top) and frequency and numbers (bottom) of B cells, T cells, $CD4^+$ and $CD8^+$ T cells as well as $CD44^+$ and $CD62L^+$ T cells

(B) Treg populations in the tissues of $CD1d^{+/-}$ and $CD1d^{-/-}$ mice showing frequency and numbers of Treg and iTreg populations

(C) C57BL/6 WT mice were orally gavaged with αGalCer and the Treg populations were analysed 3 days later in spleen. Flow cytometry plots (left) and frequency of Treg and iTreg (right) are depicted.

(D) C57BL/6 WT mice were orally gavaged with αGalCer +/- αIL-4 or αTGF-β blocking antibodies and the iNKT cell population was analysed 3 days later in mLN. Numbers show percentage of iNKT cells. Data represent 3 experiments

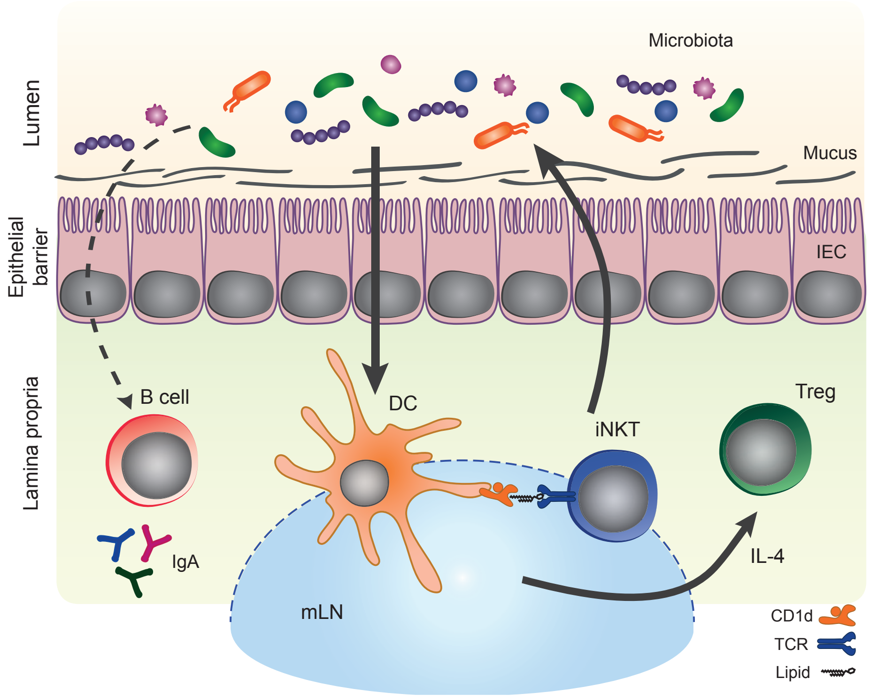

Appendix Figure S12. Proposed model for the role of CD1d and NKT cells in the intestinal homeostasis.

CD1d and NKT cells participate in the homeostatic communication between the microbiota and the intestinal immune system. Intestinal NKT cells sense lipids presented by $CD11c^{+}$ cells and this regulates NKT cell homeostasis and activation. CD1d-mediated crosstalk between NKT cells and DC cells contribute to shape intestinal commensal communities and IgA repertoire and modulate intestinal Tregs.

Table I. Antibodies used for flow cytometry

Antibody	Clone	Supplier
CD45R/B220	RA 3-6B2	BioLegend
CD1d	1B1	Biolegend
CD3ε	154-2c11	BioLegend
CD4	GK1.5	BioLegend
CD5	53-7.3	BioLegend
CD8α	56-6.7	BioLegend
CD11b	M1/70	BioLegend
CD11c	N418	Biolegend
CD19	1D3	BD Pharmingen
CD21/CD35 [CR2/CR1]	7E9	Biolegend
CD23	B3B4	Biolegend
CD24	M1/69	Biolegend
CD43	S7	BD-Pharmingen
CD44	IM7	eBioscience
CD45	104	BioLegend
CD49d	R1-2	BioLegend
CD62L	MEL-14	Biolegend
CD69	H1.2F	Biolegend
CD95	Jo2	BD Pharmingen
CD103	E27	Biolegend
CD304 [Neuropilin 1]	3E12	Biolegend
E4BP4	S2M-E19	eBioscience
Foxp3	FJK-16S	eBioscience
GL-7	GL7	Biolegend
I-A/I-E [MHC-II]	M5/114.15.2	Biolegend
IgA	Polyclonal	Southern Biotech
IgD	11-26c.2a	BioLegend
IgG1	RMG1-1	Biolegend
IgG2b	Polyclonal	Abcam
IgM	RMM-1	Biolegend
Ly-51 [BP-1]	6C3	Biolegend
NK1.1	PK136	eBioscience
Nur77	12.14	eBioscience
PD-1	29F.1A12	BioLegend
PLZF	9E12	Biolegend
ROR gamma (t)	Q31-378	BD Horizon
T-bet	4B10	Biolegend
TCRβ	H57-587	Biolegend

Table II. Primers used for qPCR

**GAPDH-F**	5' ACGACCCCTTCATTGAC 3'
**GAPDH-R**	5' TCCACGACATACTCAGCAC 3'
**Nur77-F**	5' CGGACAGACAGCCTAAAAGG 3'
**Nur77-R**	5' TAACGTCCAGGGAACCAGAG 3'
**IL-4-F**	5’ AAGAACACCACAGAGAGTGAGCTC 3’
**IL-4-R**	5’ TTTCAGTGATGTGGACTTGGACTC 3’
**IL6-F**	5' CAGAGGATACCACTCCCAACA 3'
**IL6-R**	5' TCCAGTTTGGTAGCATCCATC 3'
**IL10-F**	5' AGAAGCATGGCCCTGAAATCAAGG 3'
**IL10-R**	5' CTTGTAGACACCTTGGTCTTGGAG 3'
**IL-12p35-F**	5’ AGTTTGGCCAGGGTCATTCC 3’
**IL-12p35-R**	5’ TCTCTGGCCGTCTTCACCAT 3’
**IL-12p40-F**	5’ CATGGAGTCATAGGCTCTGGAAA 3’
**IL-12p40-R**	5’ TGGCCAGCATCTAGAAACTCTTTG 3’
**IL-17A-F**	5’ AGCAAGAGATCCTGGTCCTGAA 3’
**IL-17A-R**	5’ CATCTTCTCGACCCTGAAAGTGA 3’
**IL23p19-F**	5’ CAGCAGCTCTCTCGGAAT 3’
**IL23p19-R**	5’ ACAACCATCTTCACACTGGATACG 3’
**IFN-g-F**	5’ GCCATCAGCAACAACATAAGCGTC 3’
**IFN-g-R**	5’ CCACTCGGATGAGCTCATTGAATG 3’
**Tgfb1-F**	5’ GCAACATGTGGAACTCTACCAGA 3’
**Tgfb1-R**	5’ GACGTCAAAAGACAGCCACTCA 3'
**Csf2-F**	5’ TGGGCA TTGTGGTCT ACAGC 3’
**Csf2-R**	5’ GCGGGTCTGCACACATGTTA 3’
